# SFTSV NSs interacts with AGO2 to regulate the RNAi pathway for viral replication

**DOI:** 10.1128/jvi.02205-24

**Published:** 2025-02-27

**Authors:** Nasir Javaid, Tae-Won Jang, Yuting Fu, Younho Choi

**Affiliations:** 1 Florida Research and Innovation Center, Cleveland Clinic 587918 , Port St. Lucie, Florida, USA; University of Kentucky College of Medicine, Lexington, Kentucky, USA

**Keywords:** SFTSV, nonstructural protein, RNAi, viral replication, bunyavirus

## Abstract

RNA interference (RNAi) is a posttranscriptional gene silencing mechanism acting as an antiviral defense in eukaryotes. During viral replication, intermediate double-stranded RNAs are processed into virus-derived small interfering RNAs (vsiRNAs) by the host enzyme, DICER. These vsiRNAs are incorporated into the RNA-induced silencing complex (RISC), where AGO2 cleaves viral genomic RNAs. However, viruses have evolved mechanisms to suppress this pathway. Here, we report that the nonstructural protein (NSs) of severe fever with thrombocytopenia syndrome virus (SFTSV) interacts with RISC to suppress the RNAi pathway. NSs forms a ternary complex by interacting with both DICER and AGO2 of the RNAi pathway. The interaction between NSs and DICER, facilitated by the two RNase III domains of DICER, is disrupted in the absence of AGO2 or the DICER-interacting domain (PIWI) of AGO2, indicating a direct interaction between NSs and AGO2. Functional assays using shRNA- and siRNA-mediated silencing of GFP signal, along with co-localization studies, demonstrated that NSs competes with siRNA to interact with AGO2, thereby abolishing RNAi activity. Mutational analysis identified an NSs-A26 mutant that no longer interacts with AGO2 and is unable to suppress RNAi activity, suggesting that NSs acts as a viral suppressor of RNAi (VSR) for SFTSV. Viral infection led to the generation of vsiRNA and showed higher replication in *AGO2*^-/-^ cells compared to wild-type (WT) cells, confirming the antiviral role of the RNAi pathway against SFTSV infection. These data suggest that the NSs–AGO2 interaction suppresses RNAi, counteracting the antiviral RNAi pathway, thereby facilitating SFTSV infection and pathogenesis.

**IMPORTANCE:**

RNA interference (RNAi) is the main antiviral defense pathway in plants and insects but is not predominant in mammals. While RNAi’s role in countering severe fever with thrombocytopenia syndrome virus (SFTSV) infection has been studied in ticks, its role in humans is unknown. The SFTSV nonstructural protein (NSs) forms inclusion bodies (IBs) in the host, sequestering multiple antiviral proteins and facilitating pathogenesis, contributing to SFTSV’s high mortality rate. Our study found that SFTSV NSs directly interacts with AGO2, a key RNAi protein, hindering its function. A novel NSs mutant failed to interact with AGO2 and lost its RNAi suppression ability, highlighting NSs as a viral suppressor of RNAi (VSR). Infection studies confirmed the RNAi pathway’s critical role in combating SFTSV infection. This study demonstrates NSs’s role in viral infection and suggests potential therapeutic approaches.

## INTRODUCTION

Severe fever with thrombocytopenia syndrome (SFTS) is a tick-borne infectious diseases caused by severe fever with thrombocytopenia syndrome virus (SFTSV), recently renamed as Bandavirus dabieense from the *Phenuiviridae* family of the *Hareavirales* order (1). Transmission of SFTSV from ticks to humans, primarily by the *Haemaphysalis longicornis* tick, is considered the main route of infection (2). This tick has also been found in 20 states of the Unites States, increasing the risk of SFTSV transmission (3). SFTSV infection can result in a fatality rate of 12%–50% (4). Due to its high fatality rate and the absence of vaccine and treatment, the World Health Organization has classified SFTS as one of the top 10 infectious diseases requiring urgent research attention (5).

SFTSV is a negative-sense, single-stranded RNA virus with a genome consisting of three segments: large (L), medium (M), and small (S). The L segment encodes the RNA-dependent RNA polymerase (RdRP), which is responsible for replication and transcription. The M segment encodes two envelope glycoproteins (Gn and Gc) that mediate the attachment and fusion of the virus with host cells. The S segment transcribes into nucleoprotein (N) and nonstructural protein (NSs) using an ambisense coding strategy (6). NSs is a major virulence factor for SFTSV as it forms inclusion bodies (IBs) that sequester multiple host proteins, including retinoic acid-inducible gene I (RIG-I), TRIM25, TBK1, IKKε, IRF3/7, and STAT1/2, thereby suppressing the type I interferon (IFN) signaling pathway (7). Additionally, NSs participates in immune regulation by targeting the tumor progression locus 2 (TPL2) pathway, which induces immunosuppression by promoting the production of anti-inflammatory cytokine IL-10, and the Nrf2 pathway, which promotes antioxidant responses in SFTSV infection (8, 9).

RNA interference (RNAi) is a critical biological process in eukaryotes, wherein small RNA molecules neutralize targeted mRNA molecules, thereby inhibiting translation or gene expression. RNAi also serves as an antiviral defense response in plant (10), mosquitos (11), and *Drosophila melanogaster* (12). In the RNAi pathway, the DICER recognizes a viral double-stranded RNA (dsRNA) and processes it into multiple small interfering RNA (siRNA) duplexes, typically 21 nucleotides long. The core protein of RNA-induced silencing complex (RISC), AGO2, then recognizes the guide strand of siRNA and cleaves complementary viral RNA through endonucleolytic activity (13). Mouse cells infected with encephalomyocarditis virus (EMCV) or Nodamura virus (NoV) accumulate viral siRNA (vsiRNA), a product of DICER processing (14). Similarly, human cells infected with influenza virus also exhibit the participation of the antiviral RNAi pathway (15). However, various viruses have evolved viral suppressor of RNAi (VSR) to counter the host’s RNAi-mediated antiviral defense. For example, the Tat of HIV-1 (16), VP35 of Ebola virus (17), the capsid protein of hepatitis C virus (HCV) (18), the NS1 of influenza virus (19), and the capsid protein of Zika virus (20) exhibit intense suppressor activity toward the RNAi pathway.

A recent *in vivo* study highlighted the role of the RNAi pathway in the vector, *H. longicornis* tick in preventing infection by SFTSV (21). However, it remains unclear how human RNAi pathway interacts with SFTSV and contributes to the pathogenesis of SFTSV infection. In this study, we identified SFTSV NSs as a potent VSR by interacting with components of the RNAi pathway, particularly AGO2, and elucidated the role of RNAi in the pathogenesis of SFTSV.

## RESULTS

### SFTSV NSs interacts with DICER through RNase III domains

**Fig 1:**
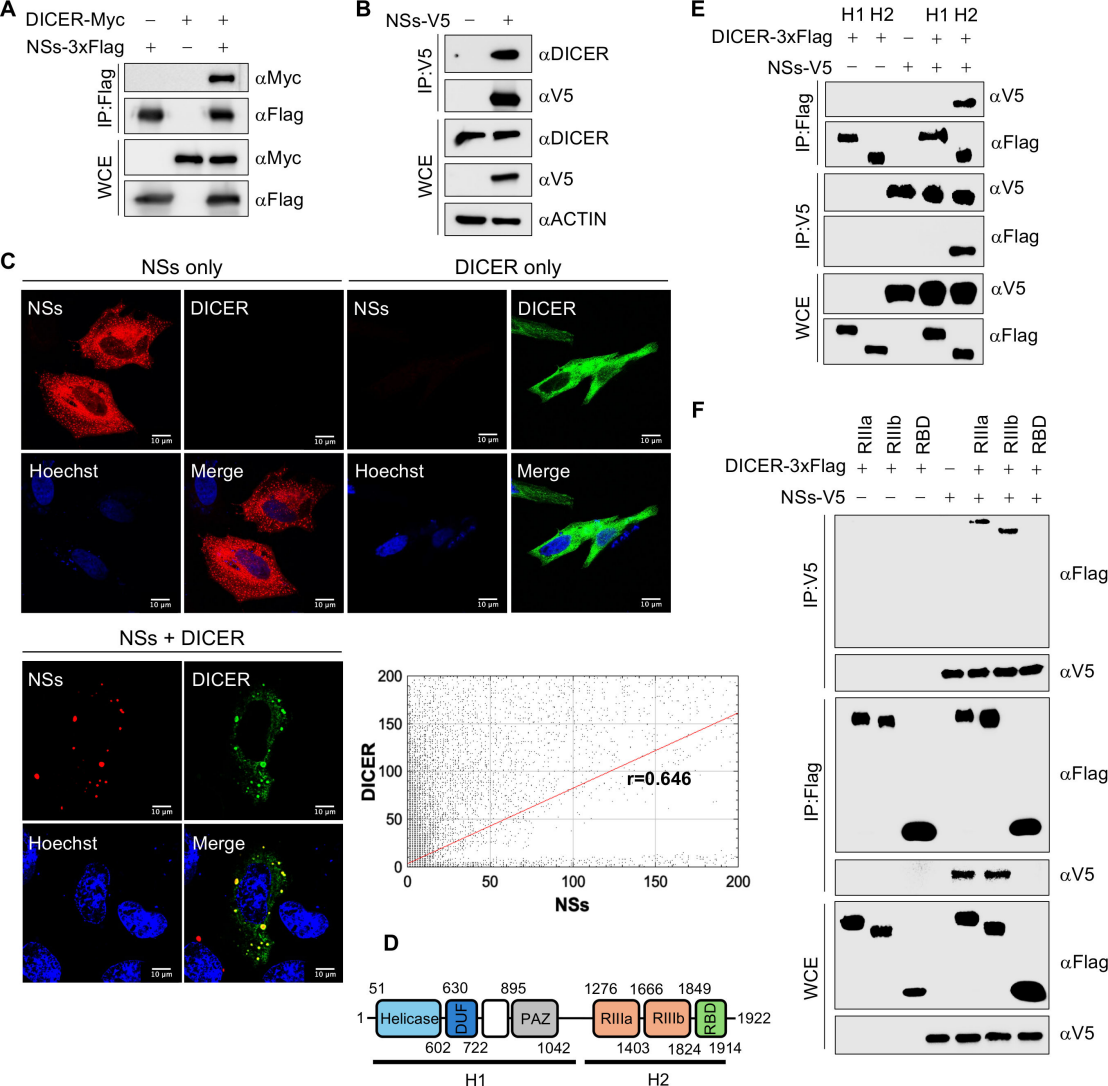
SFTSV NSs interacts with human DICER. (**A, B**) Co-IP interaction after 48 h of transfection for (**A**) overexpressed NSs-3xFlag with overexpressed DICER-Myc in HEK293T cells or (**B**) overexpressed NSs-V5 with endogenous DICER in BJAB cells. (**C**) Confocal microscopy of NSs-V5 (Alexa Fluor 647) and DICER-Myc (Alexa Fluor 488) in HeLa cells after 48 h of transfection. Scale bar, 10 µm. (**D**) Domain mapping of human DICER. (**E, F**) Co-IP interaction between overexpressed NSs-V5 and (**E**) H1 and H2 3xFlag-tagged segments or (**F**) RNaseIIIa, RNaseIIIb, and RBD 3xFlag-tagged domains of DICER after 48 h of transfecting HEK293T cells. Co-IP membranes were visualized with ChemiDoc touch imaging system (Bio-Rad). Cells were visualized with Stellaris 8 confocal microscope and were processed with Imaris software.

The complexes of SFTSV NSs were isolated from BJAB cells and analyzed via mass spectrometry, revealing DICER and AGO2 as the host-binding protein to NSs (Data Set S1). The interaction between NSs and DICER was further confirmed through co-immunoprecipitation (co-IP). Pulldown experiment using transiently overexpressed 3xFlag-tagged NSs (NSs-3xFlag) demonstrated its interaction with overexpressed Myc-tagged DICER (DICER-Myc) in HEK293T cells (Fig. 1A). Similarly, pulldown experiment using transiently overexpressed V5-tagged NSs (NSs-V5) revealed an interaction with the endogenous DICER in BJAB cells (Fig. 1B). To investigate their spatial arrangement, NSs-V5 and DICER-Myc proteins were co-expressed in HeLa cells through transient transfection, showing co-localization within the cytoplasm and sequestration of DICER into NSs inclusion bodies (NSs-IBs). The extent of co-localization is shown as cytofluorogram with Pearson’s correlation coefficient (PCC) r value of 0.646 (Fig. 1C). DICER comprises several domains, including helicase, DUF283 (domain of unknown function), PAZ (Piwi–Argonaute–Zwille), two tandem RNase III domains (RIIIa and RIIIb), and a double-stranded RNA-binding domain (dsRBD) (Fig. 1D). To identify the interacting domain within DICER, the protein was initially truncated into two segments, H1 and H2 (Fig. 1D), which then tagged with 3xFlag and overexpressed in HEK293T cells along with NSs-V5. The pulldown of H1 and H2 segments with NSs revealed an interaction specifically with the H2 segment of DICER, which comprises the RNase IIIa, RNase IIIb, and dsRBD domains (Fig. 1E). To further delineate the interacting domains, the domains in H2 segment were individually tagged with 3xFlag and overexpressed in HEK293T cells along with NSs-V5. The pulldown of these tags demonstrated the interaction of NSs with RNase IIIa and IIIb domains of DICER (Fig. 1F). These findings underscore the interaction of SFTSV NSs with DICER through its RNase III domains.

### SFTSV NSs interacts with AGO2

**Fig 2:**
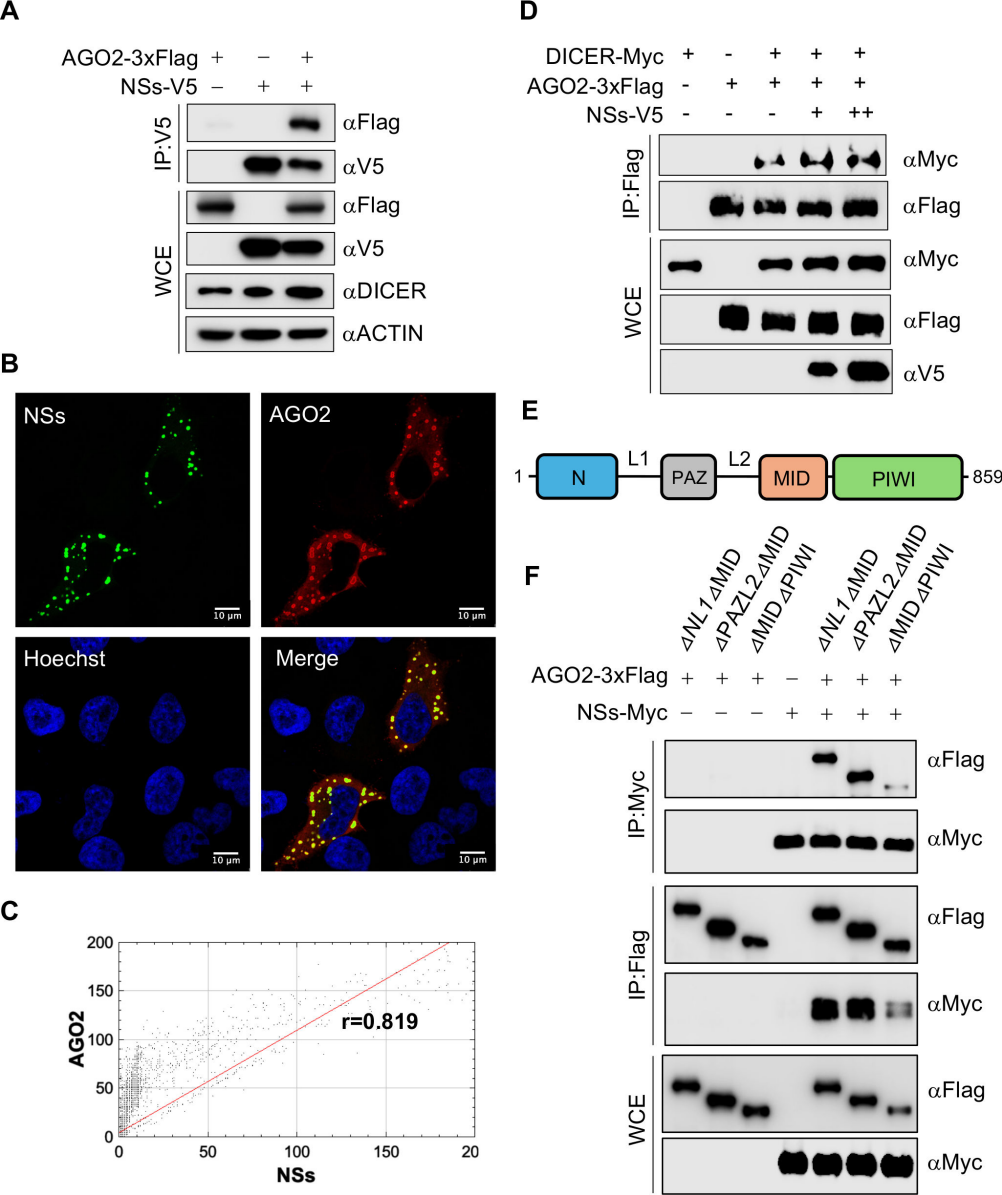
SFTSV NSs interacts with human AGO2. (**A**) Co-IP interaction between AGO2-3xFlag and NSs-V5 after 48 h of transfection in HEK293T cells. (**B, C**) Confocal microscopy to show the (**B**) co-localization of GFP-NSs and AGO2-3xFlag (Alexa Fluor 647) in HeLa cells along with the (**C**) cytofluorogram and PCC r value. (**D**) Interaction between AGO2-3xFlag and DICER-Myc in the absence or presence of NSs-V5 through co-IP of HEK293T-transfected samples. (**E**) Domain mapping of the human AGO2. (**F**) Double-domain deleted mutants of AGO2 with 3xFlag tags and NSs-Myc were analyzed for co-IP in *AGO2^-/-^* HEK293T cells. Co-IP membranes were visualized with ChemiDoc touch imaging system (Bio-Rad). Cells were visualized with Stellaris 8 confocal microscope and were processed with Imaris software. Cytofluorograms and PCC were made by JACoP plugin in ImageJ software. Scale bar, 10 µm.

AGO2 plays a pivotal role in the RNAi pathway, facilitating the cleavage of target RNA mediated by siRNAs or miRNAs (22). To investigate the interaction between NSs and human AGO2, both proteins were transiently overexpressed into HEK293T cells, each tagged with V5 and 3xFlag, respectively. Co-IP experiments revealed their mutual interaction (Fig. 2A). Subsequently, the spatial arrangement was assessed by co-expressing GFP-tagged NSs (GFP-NSs) and AGO2-3xFlag proteins in HeLa cells, demonstrating their co-localization within the cytoplasm (Fig. 2B). Cytofluorogram showed correlation in fluorescence intensity with a PCC r value of 0.819 (Fig. 2C). To confirm the RNA dependency of this interaction, total cellular RNA was degraded using RNase A/T1 mixture prior to co-IP. The results show that RNA degradation did not disrupt the interaction between NSs-V5 and AGO2-3xFlag (Fig. S1). Furthermore, to explore the impact of NSs on the interaction between DICER and AGO2, all three proteins were overexpressed in HEK293T cells, each tagged with V5, Myc, and 3xFlag, respectively. The pulldown of AGO2 demonstrated that NSs does not interfere with the interaction between DICER and AGO2 (Fig. 2D). It indicates that NSs binds to a site distinct from the interacting site of DICER and AGO2, in an RNA-independent manner. This supports the formation of a ternary complex (NSs-AGO2-DICER) among them.

Human AGO2 consists of various domains, including N, PAZ, MID, and PIWI, with linkers L1 (between N and PAZ) and L2 (between PAZ and MID) (Fig. 2E) (23). We examined the region of AGO2 that is crucial for its interaction with SFTSV NSs. To this end, we constructed mutants of the AGO2 with double-domain deletions, each tagged with 3xFlag at their C-terminal. These constructs were overexpressed in HEK293T cells along with NSs-Myc for 48 h, and the samples were analyzed for co-IP. The data showed a weaker interaction of AGO2 with NSs when the MID and PIWI domains were deleted (*△*MID*△*PIWI), compared to *△*NL1*△*MID and *△*PAZL2*△*MID (Fig. 2F). These results highlight the involvement of multiple AGO2 domains in interacting with SFTSV NSs.

### AGO2 is indispensable for the formation of NSs-AGO2-DICER complex

**Fig 3:**
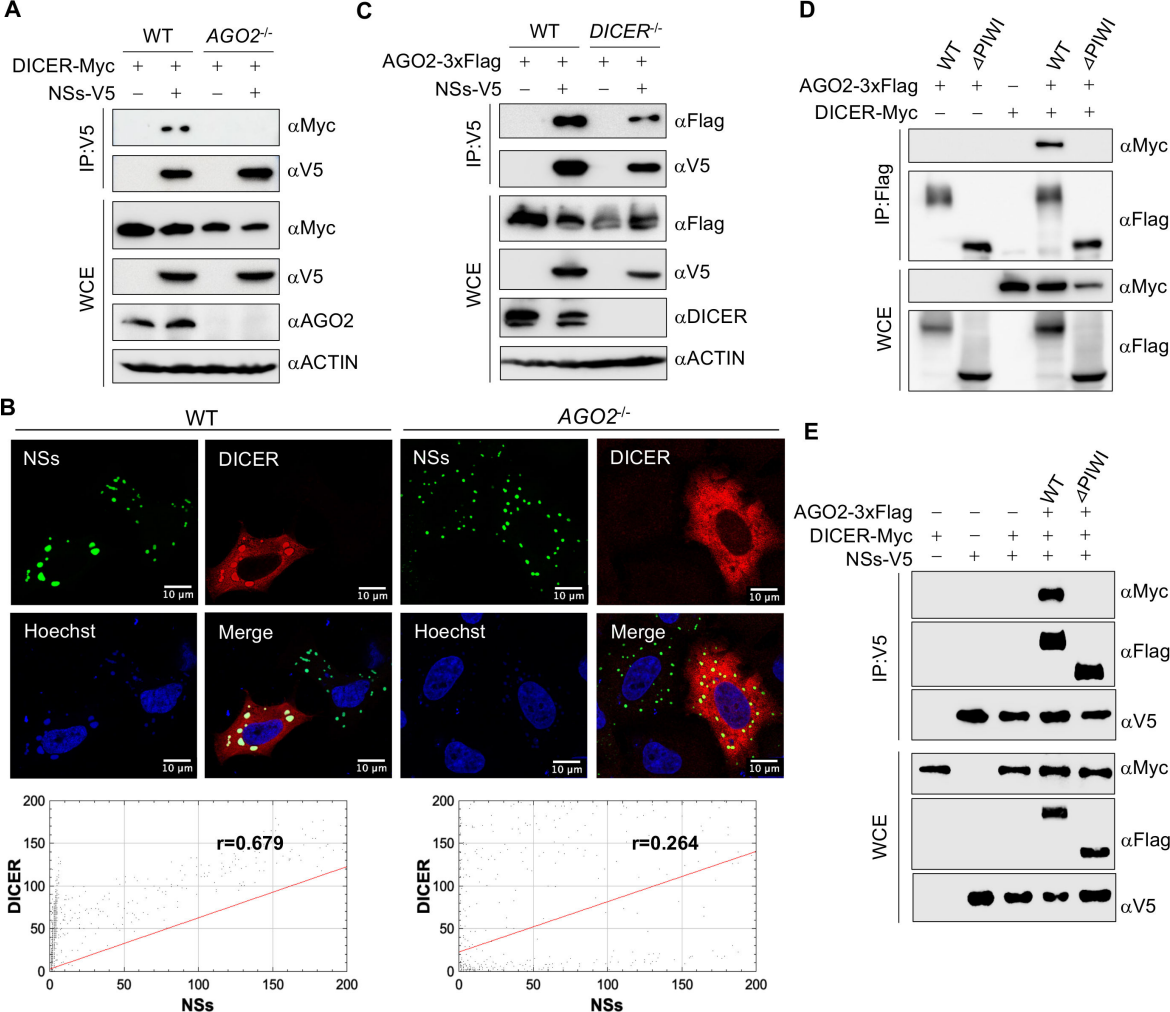
Interaction between NSs and DICER is through AGO2. (**A**) Interaction between DICER-Myc and NSs-V5 through co-IP of samples collected from WT and *AGO2*^-/-^ HEK293T cells after 48 h of transfection. (**B**) Confocal microscopy and intensity analysis between GFP-NSs and DICER-Myc (Alexa Fluor 647) after co-expressing them for 48 h in WT and *AGO2*^-/-^ HeLa cells. (**C**) Interaction between AGO2-3xFlag and NSs-V5 by co-IP of samples collected after 48 h of transfection from WT and *DICER*^-/-^ HEK293T cells. (**D, E**) Co-IP analysis from HEK293T samples for interaction between (**D**) DICER-Myc and WT or PIWI-domain deleted (*△*PIWI) AGO2-3xFlag and (**E**) DICER-Myc and NSs-V5 in the absence or presence of WT or *△*PIWI AGO2-3xFlag in *AGO2*^-/-^ HEK293T cells. Co-IP membranes were visualized with ChemiDoc touch imaging system (Bio-Rad). Cells were visualized with Stellaris 8 confocal microscope and were processed with Imaris software. Cytofluorograms and PCC were made by JACoP plugin in ImageJ software. Scale bar, 10 µm.

To further understand the interaction within the complex, wild-type (WT) and AGO2 knockout (*AGO2*^-/-^) HEK293T cells were co-transfected with NSs-V5 and DICER-Myc followed by the pulldown of NSs. The interaction between NSs and DICER was found to be lost in *AGO2*^-/-^ cells (Fig. 3A). Additionally, we observed a loss of co-localization between GFP-NSs and DICER-Myc in *AGO2*^-/-^ HeLa cells as compared to WT HeLa cells with PCC r values of 0.264 and 0.679, respectively (Fig. 3B). On the other hand, no loss of interaction was observed between NSs-V5 and AGO2-3xFlag in the absence of DICER in DICER knockout (*DICER^-/-^*) HEK293T cells (Fig. 3C). The interaction between DICER and AGO2 occurs through the PIWI domain of AGO2 (24), as also evidenced in our data using a PIWI-deleted AGO2 (AGO2-*△*PIWI) in *AGO2*^-/-^ HEK293T cells (Fig. 3D). Surprisingly, the interaction between NSs-V5 and DICER-Myc was lost when using 3xFlag-tagged AGO2-*△*PIWI compared to wild-type AGO2 (Fig. 3E). This suggests the formation of an NSs-AGO2-DICER complex facilitated by the interaction of NSs with AGO2 rather than with DICER.

The RISC comprises various components, including trinucleotide repeat-containing gene 6A (TNRC6), polyadenylate-binding protein 4 (PABPC4), and protein activator of interferon-induced protein kinase EIF2AK2 (PRKRA), which collectively participate in the repression of mRNA expression (25). We investigated the interaction of SFTSV NSs with these components. NSs-V5 showed an interaction with 3xFlag-tagged TNRC6A and PRKRA in wild-type HEK293T cells by co-IP (Fig. S2A) and a co-localization of them in wild-type HEK293T and HeLa cells by fluorescence microscopy (Fig. S2B). In *AGO2*^-/-^ cells, this interaction was lost, as observed for both the co-IP in HEK293T cells (Fig. S2C) and fluorescence microscopy in HEK293T and HeLa cells (Fig. S2D). These findings further support the central role of AGO2 in mediating the interaction between RISC and NSs.

### SFTSV NSs-A26 mutant abolishes formation of NSs-AGO2-DICER complex

**Fig 4:**
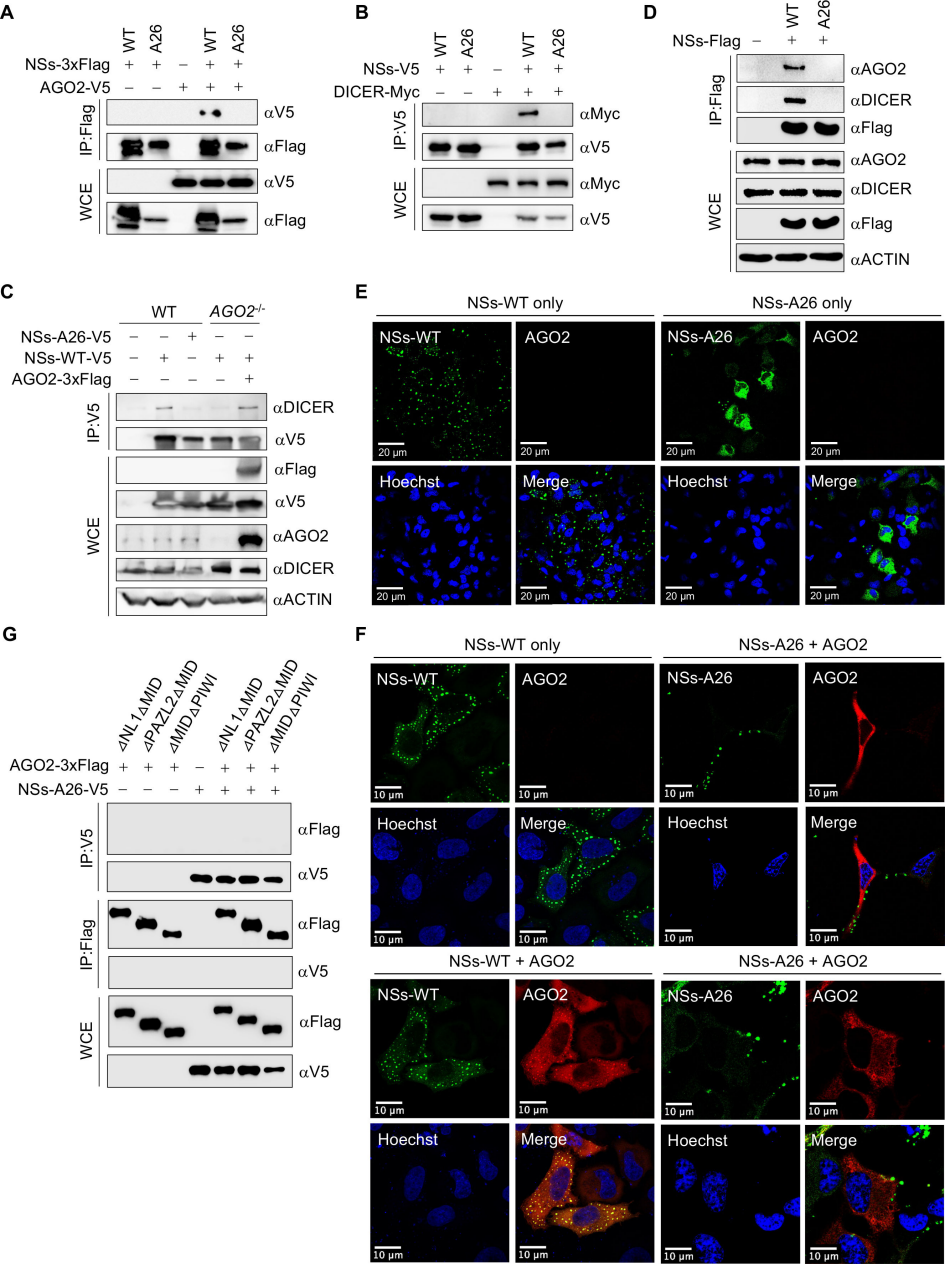
Interaction of NSs-A26 mutant with AGO2 and DICER. (**A, B**) Co-IP analysis of HEK293T samples for interaction between (**A**) AGO2-V5 and WT or A26 mutant NSs-3xFlag and (**B**) DICER-Myc and WT or A26 mutant NSs-V5 after 48 h of transfection. (**C**) Co-IP interaction between AGO2-3xFlag and endogenous DICER with the overexpression of WT or A26 NSs-V5 in WT or *AGO2*^-/-^ HEK293T cells. (**D**) Interaction between overexpressed WT or A26 NSs-3xFlag and endogenous AGO2 and DICER in BJAB cells. (**E, F**) Confocal microscopy for the (**E**) formation of inclusion bodies and (**F**) co-localization with AGO2-3xFlag (Alexa Fluor 647) by GFP-NSs-WT and GFP-NSs-A26. (**G**) Double-domain deleted mutants of AGO2 with 3xFlag tag and NSs-A26 with V5 tag were analyzed for co-IP in HEK293T cells. Co-IP membranes were visualized with ChemiDoc touch imaging system (Bio-Rad). Cells were visualized with Stellaris 8 confocal microscope and processed with Imaris software. Scale bar, 10 or 20 µm.

Based on the NSs–AGO2 interaction as a readout, we seek to identify the AGO2-binding mutant of NSs. We utilized previously developed GFP-NSs-3xFlag mutants carrying five alanine substitutions and performed co-IP with AGO2-V5 in HEK293T cells to assess interaction. Among alanine mutants, the NSs-A26 mutant carrying the replacement of 126-METGR-130 with alanine did not show interaction with AGO2 as compared to wild-type NSs (NSs-WT) in co-IP (Fig. 4A). Similarly, V5-tagged NSs-A26 did not interact with DICER-Myc compared to NSs-WT in co-IP (Fig. 4B). The V5-tagged NSs-A26 did not show interaction with endogenous DICER while V5-tagged NSs-WT showed interaction with endogenous DICER, which was also resumed after transient expression of 3xFlag-tagged AGO2 in *AGO2*^-/-^ HEK293T cells (Fig. 4C). The 3xFlag-tagged NSs-A26 also did not show interaction with endogenous AGO2 and DICER as compared to NSs-WT through co-IP in BJAB cells (Fig. 4D). Previously, we reported that SFTSV NSs interacts with TBK1 of host while NSs-P_102_A lost its interaction (8). Notably, V5-tagged NSs-A26 maintained its interaction with TBK1 unlike NSs-P_102_A mutant (Fig. S3). SFTSV NSs makes IBs in the cells, which sequester various host proteins (9). The NSs-A26 mutant showed lower IBs as compared to NSs-WT (Fig. 4E) but lost its co-localization with AGO2 (Fig. 4F). The ability of NSs-A26 to interact with AGO2 was further tested on AGO2 mutants with double-domain deletion (as shown in Fig. 2F). Overexpressed V5-tagged NSs-A26 did not show interaction with any of the 3xFlag-tagged AGO2 mutants through co-IP of samples collected after 48 h post-transfection of HEK293T cells (Fig. 4G).

### SFTSV NSs inhibits functional activity of RNAi pathway

**Fig 5:**
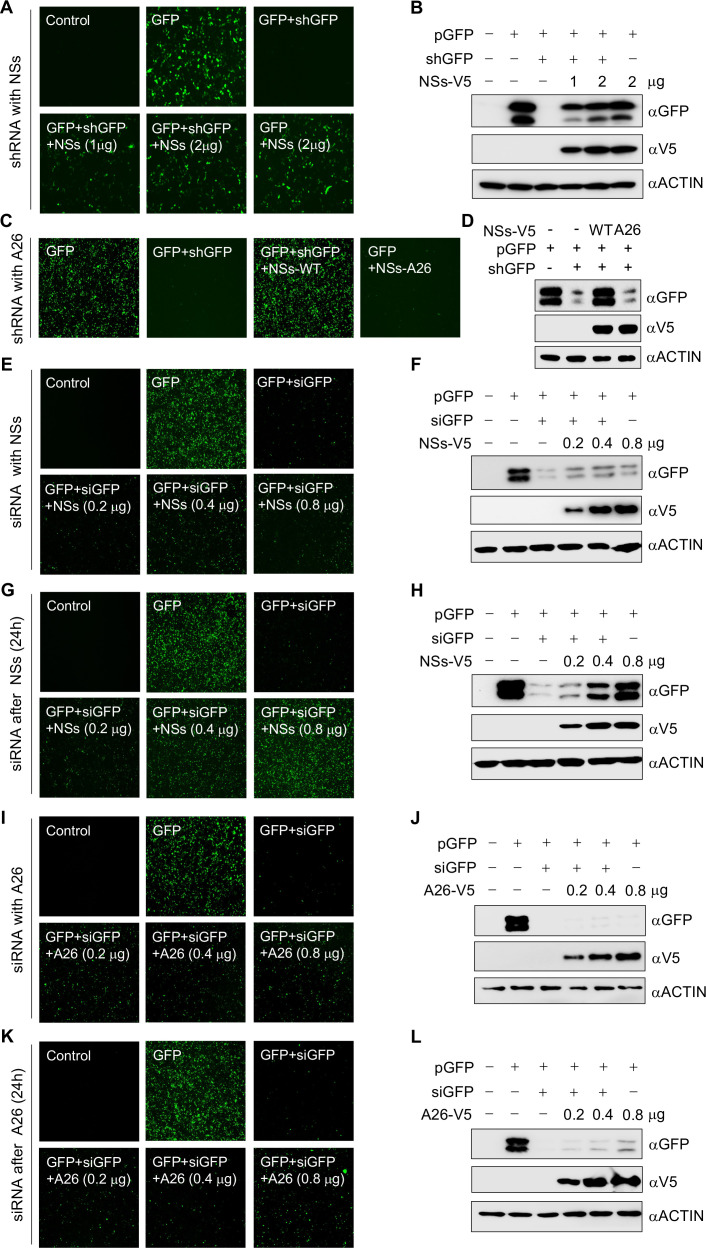
Effect of NSs on functional activity of RNAi pathway. (**A–D**) Co-transfection of pCDH-EGFP (pGFP, 200 ng), pLKO.1 GFP shRNA (shGFP, 200 ng), and (**A, B**) pIRES-NSs-V5 (NSs-V5, 1, 2 µg) or (**C, D**) pIRES-NSs-A26-V5 (A26-V5, 2 µg) plasmids into HEK293T cells in 6-well plate and analysis of GFP signal through (**A, C**) fluorescence microscopy and (**B, D**) Western blotting after 48 h of transfection. (**E–H**) Co-transfection of pGFP (100 ng), NSs-V5 (0.2, 0.4, and 0.8 µg), and siGFP (100 pmol) at (**E, F**) same time or (**G, H**) 24 h after NSs-V5 transfection into HEK293T cells in 12-well plate and analysis of GFP signal through (**E, G**) fluorescence microscopy and (**F, H**) Western blotting after 48 h of last transfection. (**I–L**) Co-transfection of pGFP (100 ng), A26-V5 (0.2, 0.4, and 0.8 µg), and siGFP (100 pmol) at (**I, J**) same time or (**K, L**) 24 h after A26-V5 transfection into HEK293T cells in 12-well plate and analysis of GFP signal through (**I, K**) fluorescence microscopy and (**J, L**) Western blotting after 48 h of last transfection. Western blot membranes were visualized with ChemiDoc touch imaging system (Bio-Rad) where actin served as a loading control. Cells were visualized by Echo Revolve inverted fluorescence microscope.

Double-stranded short-hairpin RNAs (shRNAs) exploit DICER and other components of RISC to suppress the expression of target genes via the RNAi pathway (26). HEK293T cells were co-transfected with plasmids expressing GFP (pGFP) and a short-hairpin RNA targeting GFP (shGFP) in the presence or absence of an NSs-V5-expressing plasmid at various concentrations. The shGFP reduced the expression of GFP through the RNAi pathway; however, the expression of NSs substantially restored GFP expression in a concentration-dependent manner, as observed through fluorescence microscopy (Fig. 5A) and Western blotting (Fig. 5B). Conversely, the expression of NSs-A26 did not show any recovery of GFP signal, as shown by fluorescence microscopy (Fig. 5C) and Western blotting (Fig. 5D). Single-stranded siRNAs bypass DICER and other components of RISC, directly interacting with AGO2 to form a minimal RISC complex, which effectively reduces the expression of the target gene through the RNAi pathway (27). Co-transfection of pGFP and single-stranded siRNA targeting GFP (siGFP) into HEK293T cells led to a reduction in GFP expression. Unexpectedly, co-expression of the NSs plasmid did not result in a significant recovery of GFP signal as observed through fluorescence microscopy (Fig. 5E) and Western blotting (Fig. 5F). To investigate the temporal aspects of this regulatory mechanism, we expressed NSs 24 h before co-transfecting siGFP and pGFP. Interestingly, prior expression of NSs restored GFP expression from suppression by siGFP, as observed in fluorescence microscopy (Fig. 5G) and Western blotting (Fig. 5H), suggesting that NSs needs to pre-associate with AGO2 to suppress the RNAi pathway. As expected, NSs-A26 did not restore siGFP-mediated, reduced GFP expression under both co-expression (Fig. 5I and J) and pre-expression (Fig. 5K and L) conditions. These findings support the functional inhibition of the RNAi pathway by temporal expression of NSs.

### Pre-existing siRNA can influence the interaction between SFTSV NSs and AGO2

**Fig 6:**
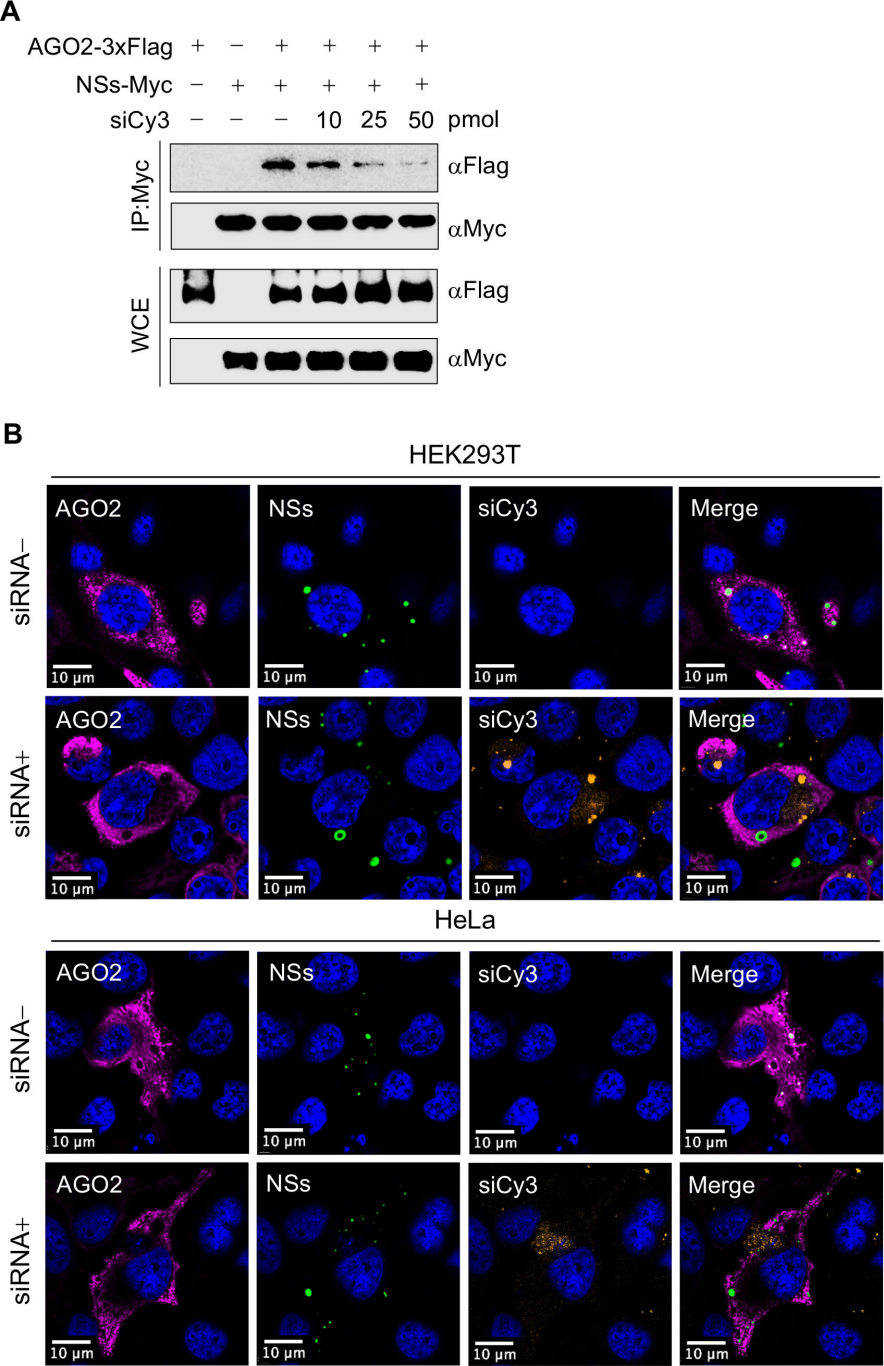
Effect of siRNA on NSs and AGO2 interaction. (**A**) *AGO2*^-/-^ HEK293T cells were transfected with pIRES-AGO2-3xFlag, pIRES-NSs-Myc, and increasing concentrations of MISSION siRNA Fluorescent Universal Negative Control #1, Cyanine 3 (siCy3) for 48 h, and co-IP was performed. Co-IP membranes were visualized with ChemiDoc touch imaging system (Bio-Rad). (**B**) Confocal microscopy of *AGO2*^-/-^ HEK293T (up) and *AGO2*^-/-^ HeLa cells (down) after 48 h overexpression of AGO2-3xFlag (Alexa Fluor 647) and GFP-NSs in the presence or absence of siCy3. Cells were visualized with Stellaris 8 confocal microscope and processed with Imaris software. Scale bar, 10 µm.

The reduction of the inhibitory effect of NSs on the RNAi pathway in the pre-existing siRNA (Fig. 5E and F) was further investigated using MISSION siRNA Fluorescent Universal Negative Control #1, Cyanine 3 (siCy3). HEK293T cells were co-transfected with either AGO2-3xFlag and NSs-Myc alone or with increasing concentrations (10, 25, and 50 pmol) of siCy3 simultaneously for 48 h. Total protein was extracted from transfected cells, and the pulldown of NSs-Myc demonstrated a decrease in the interaction between NSs and AGO2 in the presence of siCy3 (Fig. 6A). Previously, we showed through immunofluorescence that NSs and AGO2 co-localize within NSs-IBs in the cytosol. However, the co-localization of NSs with AGO2 was disrupted by the presence of siCy3 in both HEK293T and HeLa cells (Fig. 6B). Together with the results in Figure S1, these findings suggest that the interaction between NSs and AGO2 is independent of endogenous RNA; however, the active engagement of AGO2 with exogenous siRNA impedes its interaction with NSs.

### RNAi pathway interferes with SFTSV infection

**Fig 7:**
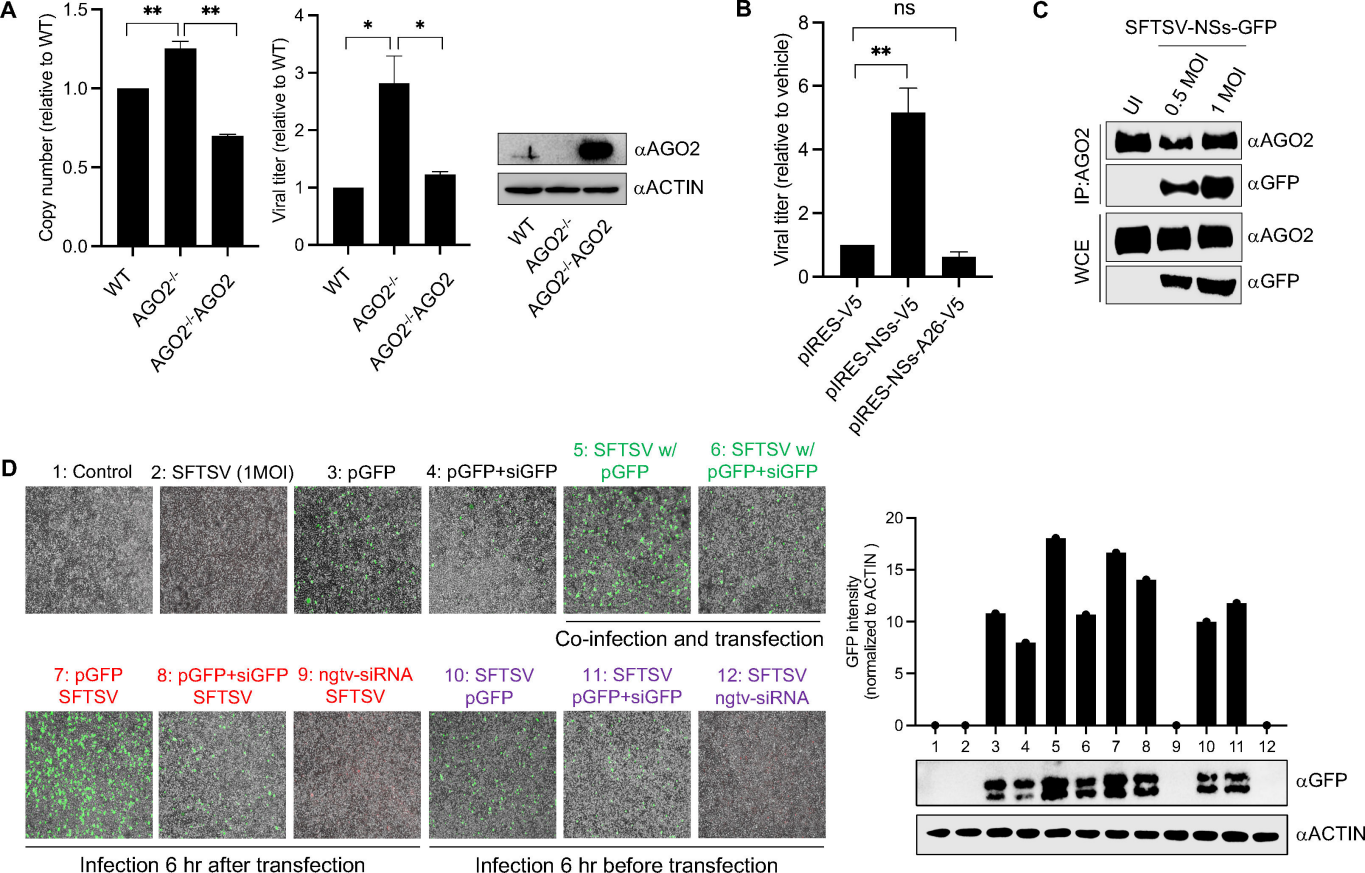
Effect of RNAi on SFTSV infection. (**A**) WT, *AGO2*^-/-^, and *AGO2*^-/-^ with trans-expression AGO2 (*AGO2*^-/-^/AGO2) HEK293T cells were infected with SFTSV (0.5 MOI), and M-segment copy number was calculated through qRT-PCR along with viral titer on Vero-E6 cells. Protein level was measured through Western blotting (*n* = 4). (**B**) HEK293T cells were transfected with pIRES-V5, pIRES-NSs-V5, or pIRES-NSs-A26-V5 plasmid for 24 h followed by SFTSV infection (0.5 MOI) for 48 h. Supernatant was analyzed for viral titer on Vero-E6 cells. Protein level was measured through Western blotting (*n* = 4). (**C**) HEK293T cells were infected with recombinant SFTSV-NSs-GFP (0.5 or 1 MOI) for 48 h; endogenous AGO2 was pulled down; and protein samples were analyzed for interaction with NSs-GFP. (**D**) HEK293T cells were infected with SFTSV (1 MOI) and co-transfected with siGFP and pGFP simultaneously, 6 h after transfection, or 6 h before transfection followed by the fluorescence microscopy for GFP signal with EVOS M5000 imaging system (Thermo Fisher Scientific). Protein samples were analyzed for GFP signal by Western blotting, and bands were quantified by ImageJ software. Scale bar, 3 µm. Bar graphs were made by using GraphPad Prism software, and statistical analysis was calculated by Student’s two-tailed *t*-test (*n* = 4).

To examine the role of AGO2 in SFTSV replication, we infected WT and *AGO2*^-/-^ HEK293T cells with 0.5 MOI of SFTSV. AGO2 deficiency (*AGO2*^-/-^) led to a higher SFTSV load in cells compared to WT, and this increase was reversed by the trans-expression of AGO2 (*AGO2*^-/-^/AGO2), as demonstrated by viral transcripts (M-segment) measured using quantitative RT-PCR (qRT-PCR) as well as viral titer (Fig. 7A). We also infected WT and *AGO2*^-/-^ HEK293T cells with 0.1 MOI recombinant SFTSV-NSs-GFP virus and compared the replication of SFTSV by analyzing GFP signal through Western blotting and fluorescence microscopy. We observed an increased GFP signal in the absence of AGO2 as compared to in its presence (Fig. S4). The role of AGO2 in SFTSV pathogenesis was further confirmed by blocking the AGO2 with the overexpression of NSs followed by infection with 0.5 MOI of SFTSV. Prior expression of NSs (but not of NSs-A26) significantly increased the titer of SFTSV (Fig. 7B) but not that of SARS-CoV-2 (Fig. S5), which supports the specific effect of SFTSV NSs on host RNAi pathway during SFTSV infection.

To assess the effect of SFTSV on the functional activity of the RNAi pathway, we first confirmed the interaction between endogenous host AGO2 and SFTSV NSs under infection condition. Recombinant SFTSV-NSs-GFP virus was infected (0.5 or 1 MOI) to HEK293T for 48 h; AGO2 was pulled down; and samples were analyzed through Western blotting. An MOI-dependent interaction between NSs and AGO2 was found in our data (Fig. 7C). Next, we infected HEK293T cells with SFTSV (1 MOI) and co-transfected with siGFP and pGFP simultaneously, 6 h after transfection, or 6 h before transfection. We observed no inhibitory effect of SFTSV infection on the activity of the RNAi pathway when infected at the same time point or 6 h after transfection. However, we noted a relatively lower activity of the RNAi pathway with SFTSV infection occurring 6 h before transfection (Fig. 7D). These findings support the idea of the antiviral RNAi pathway on SFTSV infection and suggest that AGO2 inhibits SFTSV replication, while the function of the RNAi pathway can be compromised by established SFTSV infection.

In antiviral RNAi, DICER cleaves viral dsRNA into viral siRNAs (vsiRNAs) that are loaded into a RISC. Antiviral RNAi depends on the efficient production of vsiRNAs from viral dsRNA and the efficient targeting of viral RNA by the RISC machinery (13). To understand the generation of vsiRNA in SFTSV-infected cells, we infected MM.1s cells with 0.5 MOI of SFTSV for 4 d and performed deep-sequencing of small RNAs. Notably, we found a single vsiRNA sequence from the L-segment of SFTSV genome with a predicted stem loop structure. Total length of the L-segment is 6,368 bp, and vsiRNA ranges from 6,202 to 6,246 bp (Fig. S6A). To confirm the effect of SFTSV infection on host cell, we analyzed the expression level of host miRNAs after infection and observed differential host miRNA biogenesis. It led to the identification of hsa-miR-21-5p with eightfold upregulation and another miRNA with 30-fold downregulation (Fig. S6B). It indicates some alterations in the RNA biology after SFTSV infection.

## DISCUSSION

Since its discovery, SFTSV has been a significant public concern due to the lack of available treatments. Among the viral proteins, NSs plays a critical role in SFTSV replication and is known to form IBs associated with lipid droplets (28). These IBs inhibit host antiviral IFN signaling pathways by sequestering key molecules such as TBK1/IKKε, STAT1/2, and IRF7 (7). In addition to interferon, RNAi is recognized as an important antiviral mechanism in eukaryotes, especially in insects (12), nematodes (29), plants (10), and fungi (30). The Asian longhorned ticks, a vector for SFTSV, utilizes the RNAi pathway to generate 22-nucleotide vsiRNAs (21). Although RNAi is also a functional antiviral pathway in mammals (14), the impact of SFTSV or its protein on the RNAi pathway in human cells has not been explored. This study demonstrates that the SFTSV NSs can disrupt the human RNAi pathway by interacting with its components, thereby inhibiting its antiviral function.

The RNAi pathway begins with the recognition of target dsRNA by DICER, which processes it into 21-nucleotide siRNA duplexes (31). These siRNAs are then incorporated into the RISC, which includes multiple components such as AGO2, TNRC6A, PABPC4, PRKRA, and others (25). Among these, AGO proteins play a pivotal role in RNA interference. Human cells have four AGO proteins (AGO1, AGO2, AGO3, and AGO4), with AGO2 being crucial for using the guide strand of siRNA to locate complementary RNA and subsequently cleave it (32). This process leads to the degradation and clearance of viral RNA from the cell. Our study reveals that SFTSV NSs interacts with several components of the RNAi pathway, including AGO2, DICER, TNRC6A, and PRKRA. Notably, the interaction with later proteins is dependent on AGO2, indicating that NSs interacts with multiple components of RISC primarily through AGO2. Furthermore, the direct interaction between NSs and AGO2 is RNA independent, as it persists even after RNase treatment. This mode of interaction is reminiscent of the inhibition of the RNAi pathway by other viral proteins, such as the CrPV-1A of cricket paralysis virus (33) and VP1 of Nora virus (34), both of which directly interact with AGO2. On other hand, AGO2 has also been implicated in enhancing the stability of viral RNA of HCV, through interaction involving cellular miR-122 (35), and enterovirus 71 involving the human antigen R (HuR) (36). Our data demonstrate increased SFTSV load in the absence of AGO2, highlighting the inhibitory effect of AGO2 on SFTSV replication. Typically, single-stranded RNA viruses first make dsRNA intermediates by synthesizing complementary strand. These dsRNA intermediates are recognized by the RNAi machinery of host cell to produce vsiRNAs, which are used by AGO2 of RNAi to cleave the target genome of virus. However, dsRNA intermediates are not common to be detected for negative-sense RNA viruses (37), which could be due to immediate association of nucleoprotein with newly synthesized genomic RNA (38). Despite no detection of dsRNA, vsiRNAs matching to both strands of viral genome were detected along with high titer of virus in RNAi mutants (39). Other studies detected the presence of dsRNA with the infection of negative-sense RNA viruses by using different detection techniques (40, 41). It strongly supports the existence of dsRNA, which could be below the detection limit of some techniques but above the detection limit of endogenous DICER. Other possibility could be the displacement of nucleoprotein by the helicase activity of DICER to access dsRNA. In our study, increased SFTSV load with the deletion of AGO2 also supports the involvement of the RNAi pathway in controlling the replication of SFTSV. This finding underscores the critical role of AGO2 in the RNAi-mediated antiviral response and suggests that the interaction between NSs and AGO2 is a strategic viral mechanism to subvert host antiviral defenses.

AGO2 in humans comprise multiple domains (N, PAZ, MID, PIWI) connected by linkers (L1 and L2) (23). Each domain plays a specific role in RNAi: the N domain acts as a wedge to split duplexes during RISC assembly (42); the PAZ and MID domains recognize 3′ and 5′ ends of guide strand, respectively (43–45); and the PIWI domain is responsible for endonuclease activity as well as interaction with the DICER (24). Our study found that deletion of the PIWI domain from AGO2 abolishes the ability of DICER to interact with NSs, suggesting that the interaction between DICER and NSs is mediated indirectly through the AGO2 PIWI domain. This indirect interaction underscores the critical role of AGO2 in the NSs-mediated inhibition of the RNAi pathway. This mechanism of RNAi pathway inhibition is not unique to SFTSV. The capsid protein of Zika virus (20), NS1 of influenza A virus (15), and 3A of human enterovirus 71 (46) have also been shown to inhibit the host RNAi pathway by directly interacting with or involving DICER. These findings highlight a common strategy among diverse viruses to evade host antiviral responses by targeting key components of the RNAi pathway.

The emergence of viral suppressors of RNAi (VSRs) enables viruses to establish infections in their host by blocking the RNAi pathway. Proteins such as CrPV-1A, VP1, capsid, NS1, 3A serve as VSRs in their specific host. However, the inhibitory activity of these VSRs can be disrupted by specific mutations. For instance, the ZIKV-H41R mutant of Zika virus capsid (20) and the HEV71-3A mutant of human enterovirus 71 (46) lose their ability to inhibit the RNAi pathway due to the loss of interaction with target proteins. Previously, we reported that NSs-P_102_A, NSs-K_211_R, and NSs-A46 mutants of the NSs reduce the pathogenicity of SFTSV by affecting the interaction of NSs with their host targets (8, 9). In this study, we identified a novel alanine mutant NSs-A26, which forms relatively reduced number of IBs as compared to wild-type NSs (NSs-WT). Notably, NSs-A26 lost its ability to interact with the AGO2, and IBs formed by NSs-A26 did not co-localize with the AGO2. The NSs-A26 mutant lost its interaction with AGO2 but maintained interaction with TBK1 (another host protein), which shows the retention of inhibitory role of NSs-A26 on IFN signaling like NSs-WT (8). It also indicates that SFTSV NSs interacts with various host proteins in genetically independent manner. Additionally, NSs-A26 does not interfere with the functional activity of the RNAi pathway, contrasting with NSs-WT. These findings highlight the critical role of the NSs in the suppression of the RNAi pathway and suggest that specific mutations can significantly alter the ability of SFTSV to evade host antiviral defenses. It also indicates that interaction with RNAi pathway proteins is not due to the formation of IBs but due to the presence of NSs-WT (not NSs-A26) inside those IBs. This insight provides a potential avenue for the development of antiviral strategies targeting the NSs and its interaction with RNAi pathway components.

The ability of SFTSV NSs to bind with AGO2 suggests potential disruption of the RNA pathway’s functional activity. The downregulation of target genes mediated by shRNA or siRNA involves the RNAi pathway components DICER (26) and/or AGO2 (27). In our study, co-expression of NSs with shGFP and GFP effectively inhibited the RNAi pathway, unlike siGFP in co-expression of NSs. However, pre-expression of NSs before introducing siGFP and GFP restored its inhibitory effect on the RNAi pathway. This is because the pre-existence of siRNA inside the cell hinders the interaction between NSs and AGO2 as evidenced by our co-IP and co-localization data. Furthermore, this trend in RNAi pathway inhibition was observed when NSs was replaced with SFTSV. In terms of SFTSV, pre-infection of cells probably gives virus extra time to block the RNAi pathway; however, it is not the case with co-transfection and infection as well as post-infection. These results suggest a possible inhibitory role of NSs on the RNAi pathway as AGO2 of infected cells will be blocked by SFTSV NSs, which will not allow it to block viral replication/transcription through the machinery of RNAi pathway. These findings suggest a possible competition between siRNA and NSs for binding to the AGO2, a phenomenon previously reported for other VSRs (47). This competition underscores the critical interplay between viral proteins and host RNAi components, revealing a sophisticated viral strategy to evade host antiviral mechanisms. Our data highlight the importance of timing in the expression of viral proteins and RNAi components, which could influence the effectiveness of RNAi-based antiviral therapies. It has also been reported that pre-treatment of human and mouse cells with siRNA, targeting the genome of poliovirus, significantly reduced the poliovirus titer and increased its clearance from infected cells (48). Binding of 3′-end of guide strand of siRNA to AGO rotates the MID and PAZ domains to 22° and 25°, respectively, which facilitates complete binding of guide strand due to 8 Å extension of nucleic acid binding channel (49). These conformational changes in the structure of AGO2 due to the pre-existence of siRNA inside the cell could also be responsible for hindering the binding of NSs to AGO2. Notably, we also found a relatively increased expression of reporter gene on infecting SFTSV together or after the transfection of reporter gene plasmid. It could be due to enhanced activity of host transcription and/or translation machinery in early phase of SFTSV infection due to the involvement of certain factors (50, 51). It would be interesting to further understand this phenomenon particularly with reference to SFTSV infection.

During infection, SFTSV targets the B cells, especially plasmablasts, of humans (52, 53), which could be due to the higher expression level of SFTSV receptor, C-C motif chemokine receptor 2 (CCR2) (54). Here, the expression of SFTSV NSs in human B cell line (BJAB) showed the interaction of NSs with DICER of RNAi pathway. Viral infection significantly impacts the host’s miRNA profile (55) and induces the generation of vsiRNAs via RNAi pathway (13). Our data identified a host miRNA candidate with 30-fold downregulation that is in need of further validation for its abundance, processing, and mechanism of action with reference to SFTSV infection. We also observed significant upregulation of hsa-miR-21 following SFTSV infection of B lymphoblast cell (MM.1s). The elevated levels of miR-21, which target various tumor suppressor pathways (56), are recognized as potential biomarkers for the early diagnosis of cancer (57). Infection with oncogenic (58) as well as non-oncogenic viruses (59) has been shown to increase miR-21 level, enhancing viral survival and replication in the host. Conversely, increased miR-21 levels have been found to suppress the replication of bursal disease virus by targeting VP1 (60). Further research is needed to determine the role of miR-21 in SFTSV survival and replication within the host. Our study detected a single type of vsiRNA derived from the L-segment of SFTSV genome at 4 d post-infection (dpi) in MM.1s cells. In contrast, the previous study has identified vsiRNAs in ticks primarily from the S segment after 6 dpi (21). This discrepancy suggests that further studies using better detection system and expertise along with various human cell lines at different time points post-infection are necessary to elucidate the role of the human RNAi pathway, the quantity of vsiRNA, and their origins. The relatively low amount of vsiRNA could be attributed to the action of NSs as a VSR. This is supported by evidence that VSR-deficient viruses, such as Nodamura virus (14) and HEV71 (46), produce higher levels of vsiRNAs. Future studies should focus on profiling vsiRNAs in human cells infected with NSs-deficient or NSs-A26 mutant SFTSV to better understand the interaction between viral components and the RNAi pathway. Moreover, comparing the vsiRNAs profile, under the condition of NSs-mediated AGO2 inhibition or AGO2 knockout cell line, with controls will further provide support to our findings.

Mammals generally have three antiviral systems: RNAi, innate immune system, and adaptive immune system. RNAi has been underrated due to lesser evidence of its antiviral activity in the presence of well-studied RIG-I, TLR, and cGAS antiviral pathways. It was believed so until the finding of Dicer-processed 22 nucleotide RNAs by deep sequencing of mouse cells infected with NoV and EMCV. A NoV mutant virus lacking VSR rescued the virus in the absence of functional RNAi system (14, 61). Similarly, VSR-mutant influenza virus (15) and human enterovirus 71 (46) significantly increased the production of RNAi-mediated vsiRNAs in human cells and mice. To exclude antiviral effects by interferons, interferon-deficient systems were used for wild-type and VSR-deficient viruses, which showed more production of RNAi-mediated vsiRNAs with later (46). The ability of RNAi to generate vsiRNAs and use them to control viral infection can be exploited for therapeutic potential against diseases like COVID-19 (62). For example, synthetic siRNAs are being designed against various viral or host proteins in order to control viral infection with the help of RNAi (63, 64). ALN-RSV01, an siRNA against nucleocapsid protein of respiratory syncytial virus (RSV), has been tested for its antiviral activity in humans (65). Specifically, further understanding of the role of the RNAi pathway in SFTSV infection will provide valuable insights into potential therapeutic targets and strategies for controlling SFTSV infection.

## MATERIALS AND METHODS

### Bacterial strains, cell lines, and culturing

TOP10 strain of *Escherichia coli* was grown in Luria-Bertani (LB) medium (Difco; BD Biosciences) for transformation of plasmid DNA, and the colonies were selected under appropriate antibiotics (kanamycin, 50 µg/mL; ampicillin, 100  µg/mL). BHK21-T7, Vero-E6, HEK293T, HeLa, and BJAB cells were cultured in Dulbecco’s modified Eagle’s medium (DMEM; Gibco, BRL, Grand Island, NY, USA) containing 10% fetal bovine serum (FBS; Genesee Scientific, San Diego, CA, USA) and 1% Pen/Strep solution (100 U/mL penicillin and 100 µg/mL streptomycin; Gibco). MM.1s cells were maintained in RPMI-1640 medium (Gibco) containing 10% FBS (Genesee Scientific) and 1% Pen/Strep solution (Gibco). All mammalian cells were maintained in a humidified incubator at 37°C with 5% CO_2_. DICER-knockout (*DICER*^-/-^) and AGO2-knockout (*AGO2*^-/-^) human cell lines were generously provided by collaborators (Dr. Bryan R. Cullen and Dr. Ronald Van Rij, respectively).

### Plasmids and reagents

Virally encoded cDNA was used to synthesize DNA encoding NSs of SFTSV. The cDNAs for human DICER and AGO2 were obtained from Addgene (Watertown, MA, USA) and Origene (Rockville, MD, USA). The constructs used for the expression of relative cDNAs in mammalian cells included pDEST-MCS-myc-puro, pIRES-MCS-3F-puro, pIRES-MCS-V5-puro, pCDH-MCS-puro, pLKO.1-TRC cloning vector, and pLKO.1 GFP shRNA vectors. All NSs expression plasmids contained a C-terminal 3× Flag tag or V5 tag only, or N-terminal GFP; DICER expression plasmids had C-terminal 3× Flag or Myc tag; and AGO2 expression plasmids had C-terminal V5 or 3× Flag tag. RISC proteins TNRC6A, PABPC4, and PRKRA were expressed by using the Addgene plasmids pcDNA5/FRT/TO/FLAG-HA-SBP-TNRC6A (#42044), pFRT/TO/FLAG/HA-DEST PABPC4 (#19882), and pLenti_C_Myc_DDK_IRES_Puro_PRKRA (#106113), respectively. Overlapping PCR and standard cloning protocol were followed to make mutants with substitution and deletion of residues or domains. Rescue of cloned plasmid or amplification of plasmid DNA was achieved by transforming respective vector into competent TOP10 strain of *E. coli*, getting single clone under respective antibiotic selection, and isolating plasmid DNA with GeneJET plasmid miniprep kit (Thermo Fisher Scientific Inc., Waltham, MA, USA) or QIAGEN plasmid midi kit (Qiagen Inc., Hilden, Germany) by following the manufacturer’s protocol.

### Generation of plasmid DNA constructs

The pIRES-MCS-3F-puro constructs with individual segments or domains of DICER and AGO2 were constructed by using the PCR cloning approach. Briefly, forward and reverse primers with restriction sites were designed spanning each region of interest by using the custom DNA oligos synthesis service of integrated DNA technologies (IDT, Coralville, IA, USA). The region was amplified by using the standard PCR amplification protocol with phusion high-fidelity DNA polymerase kit (Thermo Fisher Scientific Inc.) in C1000 thermal cycler (Bio-Rad, Hercules, CA, USA). Amplicons were run on 1%–2% agarose gel along with 100 bp or 1 kb DNA ladder (Promega, Madison, WI, USA), stained with labsafe nucleic acid stain (G-biosciences, St. Louis, MO, USA), and visualized with ChemiDoc touch imaging system (Bio-Rad). Correctly sized amplicon was gel purified with the geneJET gel extraction kit (Thermo Fisher Scientific Inc.) by following the manufacturer’s protocol. Purified amplicon and vector were digested by using respective restriction enzymes (NEB, Ipswich, MA, USA), gel purified, cloned by using rapid DNA ligation kit (Thermo Fisher Scientific Inc.), and transformed into competent Top10 strain of *E. coli.* Bacterial clones were screened by colony PCR with OneTaq 2X master mix with standard buffer (NEB). Correct clones were grown for plasmid isolation followed by confirmation with sanger sequencing (Genewiz, Waltham, MA, USA).

To make mutant protein with the deletion of internal domain, overlapping PCR protocol was followed with three PCR reactions. Briefly, a primer set spanning the 5′ of the deleted region was used to perform the first PCR reaction. It was followed by the second PCR reaction with the primer set spanning the 3′ of the deleted region. Reverse primer of the first reaction and forward primer of the second reaction contained overlapping flanks. Amplicons of these reactions were gel purified, annealed, and processed for the third PCR reaction by using the primer set targeting the extremes of the DNA. After PCR reaction, the amplicon was cloned and confirmed by sanger sequencing as mentioned above. The list of primers is mentioned in Table S1.

### Co-immunoprecipitation

HEK293T cells at 70% confluency were transfected in 10 cm dish or 6-well plate with respective expression plasmids by using standard polyethylenimine (PEI) method for 48 h. Cells were collected, washed with 1× phosphate-buffered saline (PBS), and lysed with 1% NP-40 lysis buffer by three rounds of freeze thaw; whole-cell extracts (WCEs) were precleared with Sepharose beads for 1 h rotating at 4°C. For RNase treatment, WCE was treated with RNase A/T1 Mix (Thermo Fisher Scientific) for 30 min at 37°C. For co-immunoprecipitation (co-IP), RNase-treated or untreated WCEs were incubated with respective antibodies at 4°C for 24 h in the presence of protein A/G agarose beads or DynaGreen Protein A/G Magnetic Beads (Thermo Fisher). Unbound proteins were removed, and beads were washed three to five times with 1% NP-40 lysis containing various concentrations of NaCl (150–500 mM). The beads were eluted in 1–2× Laemmli dye, heated for 5–10 min at 95°C, and then subjected to immunoblotting analysis.

### Immunoblotting analysis

Sepharose precleared WCEs were used to determine the protein concentration by Pierce bicinchoninic acid protein assay (Thermo Fisher Scientific). Equal amounts of proteins were resolved on SDS-PAGE gels along with precision plus protein dual color standards (Bio-Rad) and transferred to polyvinylidene difluoride membrane at 25 V for 30–45 min through semidry transfer system (Bio-Rad). Membranes were blocked for 1 h in 5% skimmed-milk Tris-buffered saline with Tween 20 (TBST; pH 8.0; Sigma) and were probed for 24 h at 4°C with primary antibodies in TBST, 5% milk, or 5% bovine serum albumin (BSA) in TBST. The primary antibodies included DICER (clone D38E7, Cell Signaling [5362]), AGO2 (clone C34C6, Cell Signaling [2897]), GFP (clone B-2, Santa Cruz [sc-9996]; clone D5.1, Cell Signaling [2956]), β-actin (clone C4, Santa Cruz [sc-47778]), Flag (for rabbit Flag, clone F7425 [Sigma {F7425}]; for mouse Flag, clone F1804 [Sigma {F1804}]), HA (for rabbit HA, clone PRB-101P [Covance]; for mouse HA, clone 16B12 [BioLegend {901513}]), V5 (for rabbit V5, clone A190 [Bethyl {A190-120A}]; for mouse V5, Invitrogen [R960-25]), and Myc (for rabbit Myc, clone Poly9063 [BioLegend]; for mouse Myc, clone 9E10 [BioLegend {626802}]). Membranes were washed three times (5 min each) with TBST; horseradish peroxidase (HRP)-conjugated secondary antibodies (anti-mouse IgG, Cell Signaling [7076] or anti-rabbit IgG, Cell Signaling [7074]) in TBST or 5% milk in TBST was applied for 1 h at room temperature and washed three times with TBST (5 min each); bands were developed with enhanced chemiluminescence (ECL) reagent (Thermo Fisher Scientific) and visualized with ChemiDoc touch imaging system (Bio-Rad).

### Immunofluorescence microscopy

HeLa (0.75 × 10^5^ cells/mL) or HEK293T (1 × 10^5^ cells/mL) cells were seeded onto glass coverslips in 12-well plate for 24 h. Cells were transfected with respective plasmid DNA and/or MISSION siRNA Fluorescent Universal Negative Control #1, Cyanine 3 (Sigma) by using TransIT-HeLaMONSTER Transfection Kit (Mirus Bio LLC, Madison, WI, USA) for HeLa cells, PEI for HEK293T, or Lipofectamine 3000 Transfection Reagent (Thermo Fisher Scientific) for siRNA. After 24 to 48 h, cells were gently washed with 1× PBS, fixed with chilled methanol for 15 min at 4°C, washed three times with 1× PBS, blocked with blocking buffer (1× PBS/5% BSA/0.3% Triton X-100) for 1 h, and treated with respective primary antibody overnight at 4°C. The next day, cells were washed three times with 1× PBS, treated with fluorochrome-conjugated secondary antibody (Thermo Fisher Scientific) for 2 h at room temperature, washed three times with 1× PBS, counterstained for 15 min with Hoechst 33342 at room temperature, washed three times with 1× PBS, and mounted on glass slide by using ProLong gold antifade mountant (Thermo Fisher Scientific). Cells were visualized with Stellaris 8 confocal microscope (Leica microsystems, imaged with a confocal microscope [Wetzlar, Germany]) and were processed with Imaris software (Bitplane, Oxford instruments). Fluorochrome-conjugated secondary antibodies included Donkey anti-Mouse IgG (H+L) Highly Cross-Adsorbed Secondary Antibody, Alexa Fluor 488 (Invitrogen, A-21202) and Goat anti-Mouse IgG (H+L) Cross-Adsorbed Secondary Antibody, Alexa Fluor 647 (Invitrogen, A-21235). Cytofluorograms and PCC r values were calculated by using JACoP plugin in ImageJ (66).

### Mass spectrometry

BJAB cells (2 × 10^5^ cells/mL) were transduced with a lentivirus-expressing NSs-Flag or a control in 6-well plates, followed by puromycin selection to generate the stable cell lines. Cells were lysed with 1% NP-40 lysis buffer containing 50 mM Tris-HCl (pH 8.0), 150 mM NaCl, 1% Nonidet P-40 (Sigma) supplemented with complete protease inhibitor EDTA-free cocktail (Roche). After centrifugation, the supernatants were mixed with a 50% slurry of FLAG M2 beads (Sigma, M8823), and the binding reaction mixture was incubated for 4 h at 4°C. The precipitates were extensively washed with lysis buffer. Proteins bound to the FLAG M2 beads were eluted and separated in a 12% of SDS-PAGE. After Coomassie Brilliant Blue staining, protein bands specific to NSs-Flag-expressing cells were excised and analyzed by mass spectrometry at Lerner Research Institute Mass Spectrometry Laboratory. In brief, the gel pieces were washed and destained in 50% ethanol and 5% acetic acid, then dehydrated in acetonitrile, and dried in a Speed-vac. They were subsequently reduced, alkylated, and digested with trypsin by adding 5 µL of 10 ng/µL trypsin in 50 mM ammonium bicarbonate, followed by overnight incubation at room temperature. The resulting peptides were extracted from the polyacrylamide in two aliquots of 30 µL 50% acetonitrile with 5% formic acid. These extracts were combined, evaporated to less than 10 µL in a Speed-vac, and then resuspended in 1% acetic acid to a final volume of approximately 30 µL for LC-MS analysis (Thermo Scientific Fusion Lumos mass spectrometry system). The data were analyzed by using all CID spectra collected in the experiment to search the human SwissProt database.

### RNAi functional assay

HEK293T cells were seeded into 12-well plate (1.5 × 10^5^ cells/mL) and grown overnight. Cells were co-transfected with pIRES-NSs-V5, pCDH-EGFP-Puro, and siGFP-RNA (5′-GGCrUACGrUCCAGGAGCGCACC-3′) by using Lipofectamine 3000 Transfection Reagent (Thermo Fisher) either at same time or with 24 h pre-transfection of pIRES-NSs-V5. After 48 h of last transfection, cells were visualized for GFP signal by using Echo Revolve inverted fluorescence microscope (Echo, San Diego, CA, USA), and protein was isolated for Western blot analysis as explained above. For the expression of A26 mutant NSs, pIRES-NSs-A26-V5 plasmid was transfected into cells instead of pIRES-NSs-V5.

### MicroRNA sequencing

MM1.s cells were infected with SFTSV at 0.5 MOI and were harvested for total RNA purification after 4 d post-infection. Isolated total RNAs were tested for quality and free of contaminants, and enriched for small RNA species including miRNAs based on column-based purification. The NEXTflex miRNA library prep kit (Bioo Scientific) was used to add specific adaptors to the 3′ and 5′ ends of the small RNA fragments, followed by reverse transcription and amplification for sequencing on an Illumina NovaSeq 6000. The reads were subject to quality control including trimming of adapter sequences and removal of reads less than 14 bp or greater than 30 bp. After quality control, the reads were mapped to the human genome (*H.sapiens*-ENSEMBL-GRCh38.r106: downloaded 22 April 2022) using miRDeep version 2.0.0.7, and gene abundance was estimated with miRDeep version 2.0.0.7. All conditions were compared to test for any genes that were differentially expressed using R version 4.0.4 and deseq2 version 1.30.1. Each individual sample was run through the miRDeep pipeline. The pipeline is able to identify known miRNAs present in the data and predict unknown miRNAs. The pipeline maps the trimmed reads to a database of known miRNAs to identify known miRNAs. In order to test for unknown miRNAs that may be present in the human genome, the reads go through a multi-step process. First, the reads are mapped to the human genome and then the local sequence is tested for a hairpin structure. The strongest unknown miRNA candidates will have a hairpin structure, a larger pile of reads from one arm of the hairpin loop and a smaller pile of reads mapping to the other arm of the hairpin structure (once referred to as the star sequence).

### Virus titration by plaque assay

Serially diluted virus (10^−1^ to 10^−6^) in growth medium was infected to monolayer of vero-E6 cells in 12-well plate and incubated for 1 h with shaking. Growth medium was removed, and cells were covered with an overlay medium containing DMEM supplemented with 2.5% FBS, 0.5% minimum essential amino acids, 0.5% sodium pyruvate, 0.5% GlutaMAX, 1% Pen/Strep, and 1.5% Avicel RC-591 NF (FMC Biopolymer, Philadelphia, PA, USA) at 37°C for 12 d. Overlay medium was removed, and cells were fixed with 10% formaldehyde in PBS for 1 h. Following fixation, cells were stained with 1% crystal violet solution in 20% ethanol for 30 min, and plaques were visualized under white light transilluminator (UVP White Light Transilluminators, TW-43, Analytik Jena, Upland, CA, USA). The titer of virus was calculated as titer (PFU/mL) = average number of plaques × viral dilution × 2.5, where 2.5 = 1 mL (1,000 µL)/400 µL.

### Viral copy number

Viral loads of SFTSV in infected cells were determined by quantifying the M segment of SFTSV through quantitative PCR. Total RNA was isolated from infected cells or supernatant by using Direct-zol RNA purification kit or Quick RNA viral kit (ZYMO, Irvine, CA, USA). RNA was treated with DNase I (Sigma), and 1 µg total RNA was reverse transcribed into cDNA by using iScript cDNA synthesis kit (Bio-Rad). The cDNA was diluted with water (1:5) and used for PCR reaction containing diluted cDNA (1 µL), M-segment forward primer (SFTS-M-F; 0.4 µL of 5 µM), M-segment reverse primer (SFTSV-M-R; 0.4 µL of 5 µM), Probe (SFTSV-M-Probe; 0.6 µL of 5 µM), SsoAdvanced Universal Probes Supermix (0.6 µL of 2X), and water (3.85 µL). The qRT-PCR analysis was carried out in CFX96 PCR machine (Bio-Rad) with thermocycler conditions: 95°C for 30 s, 95°C for 30 s, 55°C for 30 s, and 68°C for 30 s for 40 cycles. Melt curve analysis was performed at temperatures ranging from 65°C to 95°C in increments of 0.5°C for 5 s. The sequence of primers and probe is as follows: SFTS-M-F (5′-AAGAAGTGGCTGTTCATCATTATTG-3′), SFTS-M-R (5′-GCCTTAAGGACATTGGTGAGTA-3′), and SFTS-M-Probe (5′-6FAM-TCATCCTCCTTGGATATGCAGGCCTCA-TAM-3′ [where 6FAM is 6-carboxyfluorescein]). A standard curve was made by using the C_t_ value of standard with known copy number, which was used to calculate the viral copy number in samples from their respective C_t_ value.

### Biosafety

All work with infectious agents for SFTSV was performed in the biosafety level 3 (BSL3) facility at Cleveland Clinic, Florida Research and Innovation Center.

## Data Availability

All data associated with this study are included in the main text and supplemental material. Inquiries regarding materials, data, and the elaboration of methods should be addressed to the primary contact, Younho Choi (choiy5@ccf.org).

## SUPPLEMENTAL MATERIAL

Data Set S1 jvi.02205-24-s0001.xlsx Mass spectrometry data, with genes of interest highlighted.

Supplemental material jvi.02205-24-s0002.docx Figures S1 to S6; Table S1.

ASM does not own the copyrights to Supplemental Material that may be linked to, or accessed through, an article. The authors have granted ASM a non-exclusive, world-wide license to publish the Supplemental Material files. Please contact the corresponding author directly for reuse.
