# Supplementary material for: SFTSV NSs interacts with AGO2 to regulate the RNAi pathway for viral replication
Source: J Virol. 2025 Feb 27;99(4):e02205-24. doi: 10.1128/jvi.02205-24 (PMC11998505; doi:10.1128/jvi.02205-24)
Supplement: Supplemental material — Figures S1 to S6; Table S1. [file jvi.02205-24-s0002.docx]

**SUPPLEMENTARY INFORMATION**

**Supplementary Figure 1 Effect of RNase treatment on NSs and AGO2 interaction.** Co-IP interaction between AGO2-3xFlag and NSs-V5 without or after treating whole cell lysate with RNase A/T1 treatment for 30 min at 37°C. Co-IP membranes were visualized with ChemiDoc touch imaging system (Bio-Rad).

**Supplementary Figure 2 Interaction of NSs with other RNAi pathway proteins.** (A, C) Flag-tagged TNRC6A, PABPC4, and PRKRA, and V5-tagged NSs were overexpressed in (A) WT and (C) *AGO2*^-/-^ HEK293T cells for 48 h and co-IP was performed. Co-IP membranes were visualized with ChemiDoc touch imaging system (Bio-Rad). (B, D) Confocal microscopy was performed by overexpressing Flag-tagged TNRC6A and PRKRA (Alexa Fluor™ 647) with GFP-NSs into (B) WT and (D) *AGO2*^-/-^ HEK293T (left) and HeLa (right) cells for 48 h. Cells were visualized with Stellaris 8 confocal microscope and processed with Imaris software. Scale bar: 10 μm.

**Supplementary Figure 3 Interaction of NSs mutants with TBK1.** Flag-tagged TBK1and V5-tagged NSs-A26 and NSs-P_102_A (PA) mutants were overexpressed in HEK293T cells for 48 h and co-IP was performed. Co-IP membranes were visualized with ChemiDoc touch imaging system (Bio-Rad).

**Supplementary Figure 4 SFTSV replication in *AGO2^-/-^* cells.** Wild-type (WT) and *AGO2*^-/-^ HEK293T cells were infected with 0.1 MOI of SFTSV for 48 h and cells were visualized by the fluorescence microscopy for GFP signal with EVOS M5000 imaging system (Thermo Fisher Scientific). Protein samples were analyzed for GFP signal by western blotting. Scale bar: 3 μm.

**Supplementary Figure 5 Effect of SFTSV NSs on SARS-CoV-2 titer.** Human ACE2 expressing HEK293T cells were transfected with pIRES-V5, pIRES-NSs-V5, or pIRES-NSs-A26-V5 plasmids for 24 h, followed by infection with SARS-CoV-2 (0.5 MOI) for 48 h. Supernatant was analyzed for viral titer on human ACE2 expressing Vero-E6 cells by plaque assay. Protein expression was analyzed by western blotting. Bar graphs were made by using GraphPad Prism software and statistical analysis was calculated by student’s two tail t-test (n=4).


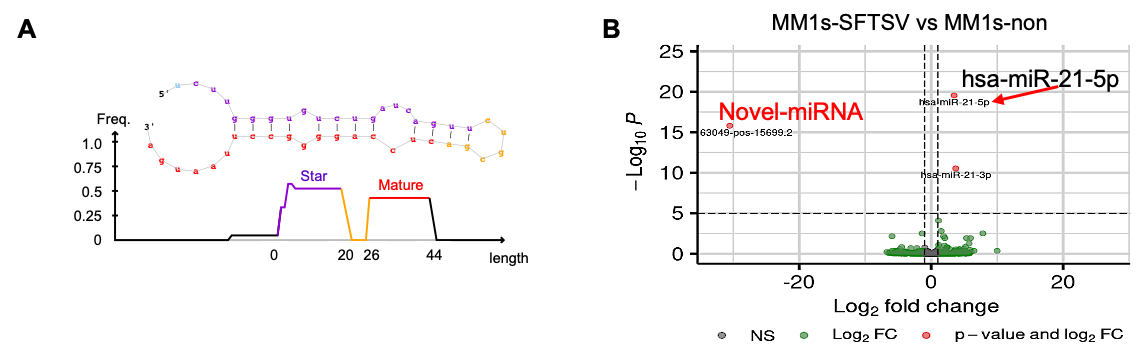


**Supplementary Figure 6 Detection of vsiRNA and host miRNA.** (A, B) MM.1s cells were infected with SFTSV (0.5 MOI) for 4 days and deep-sequencing of small RNAs was performed to analyze (A) vsiRNA and (B) miRNAs.

**Supplementary Table 1: List of primers used in this study.**

| **Construct/Reaction** | **Primer** | **5'-Sequence -3'** |
| --- | --- | --- |
| **Construct** | | |
| pIRES-Dicer-Myc-Puro | Forward | GACGCTAGCGCCACCATGAAAAGCCCTGCTTTGCAACCCCT |
|  | Reverse | AAGCTAATCAACCTCAGGTTCCCAATAGCCTCGAGATG |
| pIRES-H1-3×F-Puro | Forward | GACGCTAGCGCCACCATGAAAAGCCCTGCTTTGCAACCCCT |
|  | Reverse | CTAACAGAGACTTTTGCCAAGGAAATCTCGAGATG |
| pIRES-H2-3×F-Puro | Forward | GACGCTAGCGCCACCATGCAGCTAAATTACTACAAGCAGGAAATA |
|  | Reverse | AAGCTAATCAACCTCAGGTTCCCAATAGCCTCGAGATG |
| pIRES-RIIIa-3×F-Puro | Forward | GACGCTAGCGCCACCATGCAGCTAAATTACTACAAGCAGGAAATA |
|  | Reverse | CATCTCGAGTTCTTCAACAGCTTTGCTAGGATCC |
| pIRES-RIIIb-3×F-Puro | Forward | GACGCTAGCGCCACCATGGATGACTTTGTGGTGGGGTTCTGGAAT |
|  | Reverse | TGTACTATCCCATGATGCGGCCACTCGAGATG |
| pIRES-RBD-3×F-Puro | Forward | GACGCTAGCGCCACCATGCTAATAGAAAAGTTTTCTGCAAATGTA |
|  | Reverse | AAGCTAATCAACCTCAGGTTCCCAATAGCCTCGAGATG |
| pIRES-AGO2-3×F-Puro | Forward | GACGCTAGCGCCACCATGTACTCGGGAGCCGGCCC |
|  | Reverse | TATCGAGAATTCAGCAAAGTACATGGTGCGCA |
| pIRES-ΔNL1-3F-Puro | Forward | GACGCTAGCGCCACCATGGCACAGCCAGTAATCGAG |
|  | Reverse | TATCGAGAATTCAGCAAAGTACATGGTGCGCA |
| pIRES-ΔPAZL2-3F-Puro | Forward 1 | GACGCTAGCGCCACCATGTACTCGGGAGCCGGCCC |
|  | Reverse 1 | GCGAAGCACGCAATGGCCCACTTGTAAAACGCTGTTGCTGACACATC |
|  | Forward 2 | GATGTGTCAGCAACAGCGTTTTACAAGTGGGCCATTGCGTGCTTCGC |
|  | Reverse 2 | TATCGAGAATTCAGCAAAGTACATGGTGCGCA |
| pIRES-ΔMID-3F-Puro | Forward 1 | GACGCTAGCGCCACCATGTACTCGGGAGCCGGCCC |
|  | Reverse 1 | GGCAGCAGGATGTTGTTCACCACCTTGATCTCGATGCCCGTGTG |
|  | Forward 2 | CACACGGGCATCGAGATCAAGGTGGTGAACAACATCCTGCTGCC |
|  | Reverse 2 | TATCGAGAATTCAGCAAAGTACATGGTGCGCA |
| pIRES-ΔPIWI-3F-Puro | Forward | GACGCTAGCGCCACCATGTACTCGGGAGCCGGCCC |
|  | Reverse | TATCGAGAATTCGCCTCCCAGCTTGACGTTGATCTTCAGG |
| pIRES-ΔNL1ΔMID-3F-Puro | Primers used to make pIRES-ΔNL1-3F-Puro construct by using pIRES-ΔMID-3F-Puro as template. | |
| pIRES-ΔPAZL2ΔMID-3F-Puro | Primers used to make pIRES-ΔPAZL2-3F-Puro construct by using pIRES-ΔMID-3F-Puro as template. | |
| pIRES-ΔMIDΔPIWI-3F-Puro | It is equivalent to pIRES-NL2-3F-Puro construct. | |
